# Providing medicines in emergency and urgent care: a survey of specialist paramedics’ experiences of medication supply and views on paramedic independent prescribing

**DOI:** 10.29045/14784726.2018.12.3.3.1

**Published:** 2018-12-01

**Authors:** Adam M. Bedson, Sue M. Latter

**Affiliations:** South Western Ambulance Service NHS Foundation Trust; University of Southampton

**Keywords:** advanced paramedic, independent prescribing, paramedic, patient group directions, specialist paramedic

## Abstract

**Introduction:**

Specialist paramedics in the United Kingdom are able to undertake additional training and education in the assessment and treatment of minor illness and injuries. The provision of medication often forms a part of specialist paramedic care, but there is currently no research into the perceived usefulness or impact of the use of patient group directions or on their preparedness to undertake paramedic independent and supplementary prescribing. The aim of this study was to (a) investigate the ways in which medicines are currently supplied by specialist paramedics and (b) establish views on the introduction of paramedic independent and supplementary prescribing, including practitioner preparedness and potential impact on practice.

**Methods:**

An online questionnaire was sent to 268 specialist paramedics employed by two NHS ambulance Trusts in England who jointly employed 54% of the national population (n = 495) of specialist paramedics. Data were analysed using descriptive statistics and a framework analysis approach.

**Results:**

Patient group directions were reported to be used regularly and infections, pain and exacerbations of respiratory conditions were the most frequently treated conditions by specialist paramedics.

Although just over half of participants reported that patient group directions did not restrict their ability to supply medication to patients, a significant minority found them too restrictive. Examples of restrictions included contradictions to local antimicrobial guidance and being unable to supply sufficiently strong analgesia.

The majority of participants (66/78, 84.6%) felt confident to undertake paramedic independent and supplementary prescribing and that it would enhance both their scope of practice (70/72, 97.2%) and patient care (67/72, 93.0%). However, participants had concerns regarding organisational readiness for paramedic independent and supplementary prescribing (50/72, 69.4%), including provision of paramedic access to patient records (65/72, 90.2%) and obtaining sufficient clinical support (39/72, 54.1%).

**Conclusions:**

Patient group directions do enable specialist paramedics to supply medication to patients in order to treat a range of conditions, but at times the paramedics felt that the patient group directions restricted autonomous practice. The majority of participants felt confident to undertake paramedic independent and supplementary prescribing and anticipated that it would enhance patient care.

## Introduction

The provision of emergency and urgent care and reducing the number of patients treated in the emergency department (ED) continue to represent a significant challenge for the NHS (NHS England, 2017). Enhancements to paramedic training which enable them to treat patients in the community are therefore important (National Institute of Healthcare and Clinical Excellence, 2017a; NHS England, 2017).

Previous research has demonstrated that the initial extended paramedic role of the emergency care practitioner (ECP) which included, for the first time, the ability to supply a range of medicines using patient group directions (PGDs) resulted in significantly more patients being treated in the community than when attended by non-specialist paramedics (NSPs) (Tohira, Williams, Jacobs, Bremner, & Finn, 2014).

In the UK, the role of the specialist paramedic (emergency and urgent care) (SPEUC) has since replaced the ECP role. The College of Paramedics (CoP) has also recommended that the level of education for SPEUCs should be increased from degree to post-graduate level. Additionally, specialist paramedics who go on to develop into advanced paramedics should be educated at Master’s level (College of Paramedics, 2017).

Under current medicines legislation exemptions, all paramedics can administer a range of medicines on their own initiative to provide emergency treatment. Medicines legislation also permits NHS Trusts to authorise healthcare professionals such as SPEUCs to supply an additional range of medication such as antibiotics and analgesia, or to administer medications to treat conditions such as vomiting or acute pain, using locally-determined PGDs. These provide a legal mechanism for the supply and/or administration of a medicine to patients who meet pre-determined clinical inclusion criteria detailed within a PGD document. If these criteria are not met, however, the PGD cannot be used to legally supply or administer the medication to a patient.

Recently, independent and supplementary prescribing authority for paramedics has been approved in England and will be permissible by advanced level paramedics (Commission on Human Medicines, 2017; NHS England, 2016). At the time of this study, advanced paramedic roles had not been implemented by ambulance Trusts in England. However, freedom of information requests by the author revealed that most ambulance Trusts employed SPEUCs eligible to develop into advanced paramedic roles and adopt paramedic independent and supplementary prescribing (PISP).

However, despite the potential importance of medication underpinning the provision of community treatment by paramedics, to our knowledge, no previous research has been undertaken into either the current practice of medication supply by SPEUCs or their views regarding PISP, including its potential impact on practice. It is not known, for example, how frequently medication supply forms part of SPEUC practice, if care delivery is restricted by PGDs or whether SPEUCs feel they and their employing Trusts are prepared for prescribing roles.

The objectives of this study were therefore to:

1. Explore SPEUCs’ views regarding current practice of medication supply using PGDs, alongside any benefits or barriers to patient care from their use.
2. Ascertain SPEUCs’ views regarding individual and organisational preparedness for PISP, alongside potential benefits, drawbacks and barriers to using PISP in practice.

## Methods

The study was conducted using an online questionnaire survey. The Checklist for Reporting Results of Internet E-Surveys (CHERRIES) has been used to report the methods of this study (Supplementary 1) (Eysenbach, 2004).

A convenience sample of two large NHS ambulance Trusts in England was selected for inclusion in this study; these two Trusts jointly employed over half of the estimated national population (n = 495) of SPEUCs (286/495, 54.1%).

Inclusion criteria for the study were that participants were employed by either of the two participating Trusts as a registered paramedic or a SPEUC and were also authorised to use SPEUC PGDs. Eligible participants were identified using staff lists held by each Trust and recruited by Trust research staff by sending an invitation email which contained a participant information sheet and a link to the online survey.

An online pilot questionnaire was developed using a previously validated nurse independent and supplementary prescribing (NISP) questionnaire (Latter et al., 2010) and from researcher (AMB) insight as a practising SPEUC. The final questionnaire comprised 37 items, including a mix of closed-ended, Likert and open-ended response questions.

The pilot questionnaire was completed by eight participants (four from each participating Trust). The questionnaire was subsequently refined based on this feedback, before the final version (Supplementary 2) was sent to all eligible participants and was available for a period of 10 weeks. Both questionnaires were distributed by research and audit staff to maintain participant anonymity.

Responses to closed-ended items were entered into Microsoft Excel and analysed using descriptive statistics. Responses to open-ended questions were analysed using a framework approach to qualitative data analysis, whereby a coding framework was developed and applied to the dataset in order to identify resulting themes (Ritchie, Lewis, McNaughton Nicholls, & Ormston, 2013).

## Results

### Sample demographics

A total of 78/268 (29.0%) SPEUCs completed the questionnaire, including six participants who took part in the pilot. Of the participants, 65/78 (83.3%) held a degree level qualification and 7/78 (8.9%) had also obtained a Master’s level qualification. A further 25/78 (32.0%) were working towards a post-graduate level qualification.

### Medication supplied/administered and conditions treated by SPEUCs

**Figure 1.**
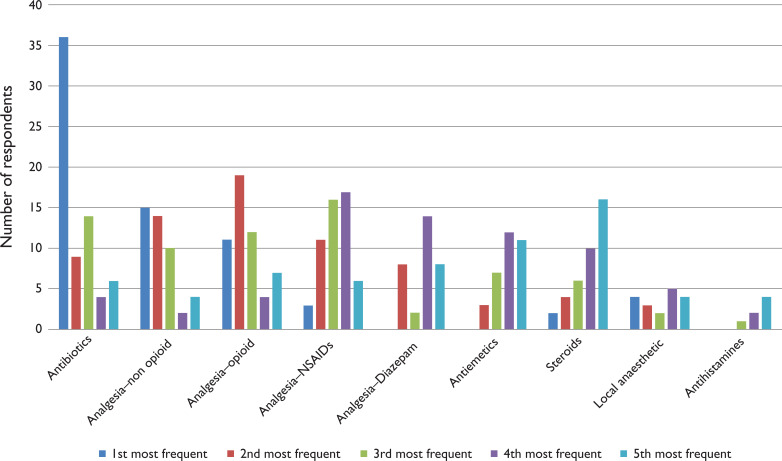
Frequently supplied/administered medication categories.

Participants reported using SPEUC PGDs regularly (Table 1), with over three quarters of the sample supplying or administering medicines on average at least once a week. Approximately one-fifth of the sample reported using PGDs an average of over three times per week. Antibiotics were the most frequently supplied medicine, followed by analgesia and steroids (Figure 1). These findings were also reflective of frequency data in the pharmaceutical usage records supplied by each Trust. The conditions frequently supplied for are shown in Table 2 and those less frequently in Table 3.

**Table 1.** Number of times PGDs used per month.

Number of times PGDs used per month	Number of respondents	% of respondents
0–3	18	23.0
4–6	18	23.0
7–9	15	19.2
10–12	11	14.1
13+	15	19.2
Question not answered	1	1.2

**Table 2.** Conditions supplied/administered for frequently.

Condition	Number of respondents who reported condition was one of the most frequently supplied/administered for	% of respondents who reported condition was one of the most frequently supplied/administered for
Lower respiratory tract infections	65	83.3
Urinary tract infections	65	83.3
Exacerbation of COPD	60	76.9
Musculoskeletal pain	57	73.0
Acute pain	54	69.2
Nausea or vomiting	36	50.0

**Table 3.** Conditions supplied/administered for less frequently.

Condition	Number of respondents who reported condition was one of the least frequently supplied/administered for	% of respondents who reported condition was one of the least frequently supplied/administered for
Infected bite wounds	58	74.0
Allergies	43	55.1
Upper respiratory tract infections	35	44.8
Chronic pain	35	44.8

### Advantages and disadvantages of PGDs

**Figure 2.**
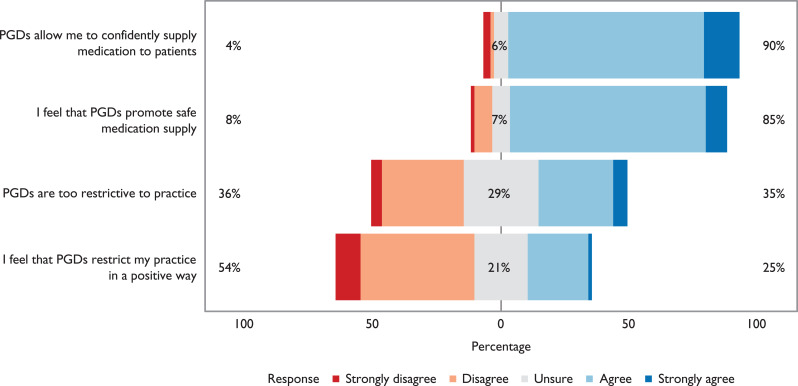
Likert rating scale data regarding the use of SPEUC PGDs in practice.

Participants were asked, using Likert rating scales, if they felt able to supply the most appropriate medication to patients using PGDs, if PGDs allowed them to confidently supply medications to patients and if PGDs promoted safe medication supply (Figure 2).

Participants also reported in open comment items that PGDs could often be used successfully and that they catered well for the range of conditions encountered. Furthermore, participants reported that despite the cost and increased work involved in maintaining stock levels, a clear benefit to using PGDs is the ability to supply medication to patients who might find it difficult to access a prescription due to being housebound or frail.

While the majority of participants felt that PGDs enabled them to confidently and safely supply medication to patients, a significant minority considered that PGDs were restrictive and did not always allow them to supply the most appropriate medication, suggesting that restrictions imposed by PGDs to ensure safe medication supply may at times inhibit autonomous practice (Figure 2).

Qualitative data also highlighted that PGD specifications did not allow the application of SPEUCs’ expertise to individual patients’ needs, and were not always sufficiently comprehensive or in line with good practice.

The PGDs are restrictive and patient co-morbidities and allergies, etc. interfere with the ideal presentation and so the PGD one size fits all doesn’t always work ... thus reducing the autonomy of the SPEUC in situations where knowledge and experience suggest benefits for a particular medication which is available but not within indications on PGD (ID 6085607027).

Closed response data revealed that 37/78 (47.4%) of participants occasionally and 16/78 (21.7%) frequently disagreed with either the inclusion or exclusion criteria of PGDs.

Participants reported disagreeing with PGD criteria most frequently when using PGDs to supply antibiotics (49/78, 62.8%), opioid analgesia (26/78, 33.3%) and steroids (24/78, 30.7%). Some participants described that antibiotic PGDs were too generic and sometimes prevented them from supplying the antimicrobial medication recommended by local prescribing guidance. Some participants also reported PGDs restricted their ability to supply sufficiently strong analgesia. For example, SPEUC PGDs only permit the supply of weak opioids such as codeine. Consequently, SPEUCs could only administer morphine (using legislative exemptions) and were prevented from supplying sufficiently strong analgesia on some occasions. As a result, it was reported that some conditions therefore needed to be escalated to a prescriber.

When asked how they overcame barriers to medication supply imposed by using PGDs, the majority of participants (66/78, 84.6%) stated they would refer the patient to a medical prescriber or would also seek a verbal order from a medical prescriber (61/78, 78.2%). Of the participants, 39/78 (50.0%) also stated they would seek a verbal order from a non-medical prescriber and 37/78 (47.4%) stated they would refer the patient to a non-medical prescriber.

Some participants considered that these processes work well and provide effective methods of overcoming PGD restrictions. Others, however, felt these processes are often unnecessary, cause delays and do not allow for the supply of controlled drugs, for which a verbal order cannot be given. Participants also highlighted difficulties in accessing medical support in order to either refer a patient who did not meet PGD inclusion criteria or to seek a verbal order where able to do so.

I think [the use of PGDs] is safer than non-medical prescribing as there is limited peer support which is difficult to access at certain times (ID 6109832319).

Exclusion criteria is frequently too restrictive for the effective management of a patient and lead to numerous hours lost waiting for GP contact to gain a verbal order, when appropriate, for the supply of medication (ID 6083204746).

Some participants commented, however, that they felt the restrictions applied by PGDs had a positive impact on practice. Most of these comments described how PGDs allowed for very safe practice and protected patients.

Although autonomy is a component of SPEUC operation, inc/exclusions on PGDs are a useful reminder of the limits of some treatments, and in the occasional case of the patient’s best interests being hindered by these, bypassing them using GP or consultant referral can be a safe alternative (ID 6084534565).

Some participants commented that PGDs were well suited to the SPEUC role based on current level of knowledge and training.

I feel our assessment skills allow me to work safely within my scope of practice and the PGDs reflect this (ID 6086532410).

I feel that currently with the pharmacological training given to SPEUCs PGDs are an appropriate means of supplying drugs to specific patient sets (ID 6071601665).

Participants also described how PGDs provided a safe method of medication supply for newly qualified SPEUCs as they gained confidence in the role but that more senior staff should be afforded more autonomy through the introduction of PISP.

PGDs are a great and safe starting point, but as soon as one develops there is the need to move to very broad PGDs, which removes the point of them and progresses towards independent prescribing (ID 6082124689).

This plethora of patient needs and medical conditions requires a judicial yet well informed practitioner who is able to have broader prescribing powers than current PGDs allow (ID 6136023209).

### Views on PISP

The majority (64/78, 82.0%) of participants appeared to be very interested in undertaking PISP training. When asked how likely they would be to undertake PISP training, 39/78 (50.0%) replied definitely yes and a further 25/78 (32.0%) replied highly likely.

The majority of participants (59/72, 81.9%) agreed with recommendations regarding eligibility for PISP and felt only existing SPEUCs rather than other paramedics who had not trained and practised as an SPEUC should be eligible to train as PISPs.

The majority of participants felt their current level of knowledge was sufficient in history taking (65/78, 83.3%), patient assessment (58/78, 74.3%) and in their ability to make a diagnosis (57/78, 73.0%) to undertake PISP training. A significant minority (14/78, 17.9%), however, reported they did not feel their ability to make a diagnosis was sufficient. Nonetheless, the majority of participants (66/78, 84.6%) agreed or strongly agreed that they would feel confident to prescribe independently following the required training.

**Figure 3.**
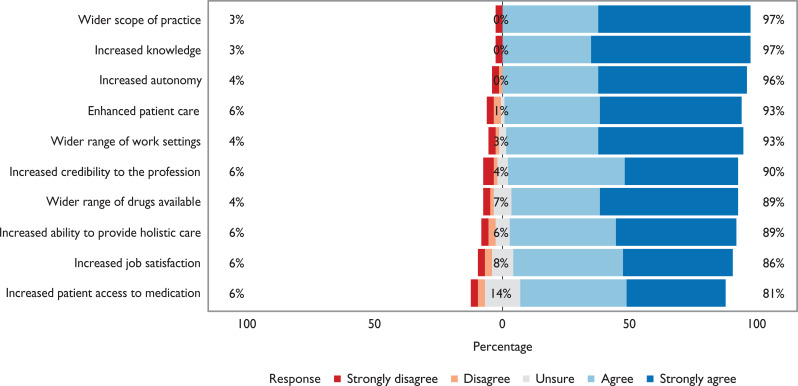
Participant responses to the question: Which of the following do you consider might be the benefits of PISP? (n = 72 – data not collected during pilot questionnaire)

**Figure 4.**
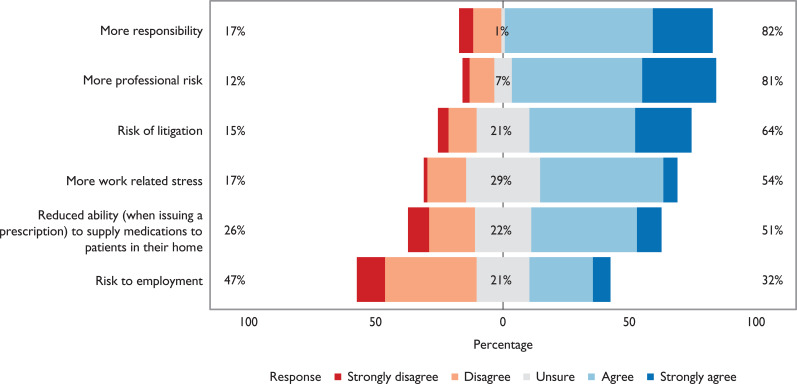
Participant responses to the question: Which of the following do you consider might be the drawbacks to PISP? (n = 72 – data not collected during pilot questionnaire)

**Figure 5.**
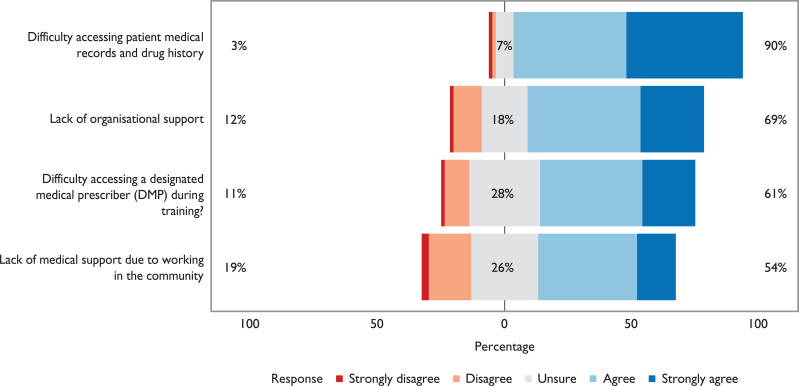
Participant responses to the question: Which of the following do you consider might occur if PISP was introduced? (n = 72 – data not collected during pilot questionnaire)

Participants were asked to indicate to what extent they agreed with a number of potential benefits and drawbacks associated with PISP. These results illustrate that the majority of participants felt PISP would lead to a wider scope of practice, increased knowledge and increased autonomy, and would enhance patient care and enable them to work in a wider range of practice settings (Figure 3). However, the majority of participants also felt that PISP would lead to increased risk and professional responsibility (Figure 4). The majority of participants also felt a lack of organisational support and difficulties accessing patient records would be encountered (Figure 5).

Participants anticipated that the most likely sources of support for prescribing decisions would be from a GP (74/78, 95.8%), another PISP (60/78, 76.9%), a doctor in the ED (43/78, 55.1%) or another non-medical prescriber (33/78, 42.3%).

## Discussion

The results from this study illustrate that the supply and administration of medicines form a regular part of SPEUC practice in order to treat a range of healthcare requirements in the community. The treatment of pain, infections and exacerbations of respiratory conditions at home by SPEUCs may potentially prevent ED attendance or hospital admission.

Several positive aspects of PGDs were cited, such as supporting the practice of less experienced SPEUCs and enabling the immediate supply of medication to patients. However, the results also highlighted how PGDs do not always allow for practitioner judgement in the application of medicines, despite a high degree of confidence and training. Importantly, PGDs do appear to sometimes restrict best practice when dealing with some of the most commonly reported conditions. This included PGDs not enabling SPEUCS to follow local prescribing guidance based on antimicrobial resistance patterns when supplying antibiotics. This is of concern in light of growing antimicrobial resistance. NICE lend support to this, outlining how PGDs should not jeopardise local and national strategies to combat antimicrobial resistance and healthcare-associated infections (National Institute of Healthcare and Clinical Excellence, 2017b).

SPEUCs in this study also reported being unable to autonomously supply sufficiently strong analgesia for some patients in pain. These restrictions sometimes led to delays in medicines supply, while an SPEUC sought the assistance of a prescriber. This has resource implications as well as implications for patient experience with regards to speed of access to medicines and continuity of care. It could also provoke unnecessary admission to hospital as participants also highlighted long delays and difficulties in referring a patient to a medical prescriber. Furthermore, while the use of verbal orders from a prescriber to overcome PGD restrictions is not prohibited by legislation, this practice is not supported by all NHS ambulance Trusts. Consequently, not all SPEUCs are able to overcome PGD restrictions in this way.

The results of this study illustrate that PGDs provide a method by which SPEUCs can supply and administer medication to patients in order to facilitate community treatment. While a number of restrictions associated with PGDs were highlighted by participants, PGDs are required to be rigid and inflexible to ensure patient safety and in order to comply with medicines legislation (National Institute of Healthcare and Clinical Excellence, 2017b). When developing PGDs, however, NHS Trusts are advised to consider the views of relevant stakeholders, including clinicians and local medicines decision-making groups (National Institute of Healthcare and Clinical Excellence, 2017b). Additionally, some less experienced SPEUCs reported that the restrictive nature of PGDs promoted safe clinical practice as they gained confidence and experience in using the additional range of SPEUC medications. The findings from this study do however emphasise the benefits that could arise from the development of SPEUCs into advanced paramedics capable of PISP. Indeed, NICE outlines that NHS Trusts should consider investing in the training of additional non-medical prescribers as an alternative to using PGDs (National Institute of Healthcare and Clinical Excellence, 2017b).

A strong likelihood of undertaking PISP training was reported by the majority of SPEUCs who participated in the study, alongside a high degree of reported readiness of skills and knowledge. This may be linked to the significant number of participants who had either completed or were undertaking post-graduate level education. The majority of SPEUCs in this study agreed with current policy recommendations and previous research findings that PISP should only be undertaken by experienced advanced level paramedics (Commission on Human Medicines, 2017; Duffy & Jones, 2017; NHS England, 2016). A recent study of nurse independent and supplementary prescribers (NISPs) lends further support to this, outlining how NISPs described the importance of both clinical experience and advanced clinical skills training for NISP (Abuzour, Lewis, & Tully, 2018).

Previous research has also demonstrated high levels of confidence and ability by NISPs when prescribing (Latter et al., 2010). A range of benefits and limitations to prescribing were reported and results from the study reported here indicate that a similar range of potential benefits and limitations are anticipated from the introduction of PISP (Courtenay, Carey, & Stenner, 2012; Herklots, Baileff, & Latter, 2015; Latter et al., 2010; Stenner, Carey, & Courtenay, 2012). Associated benefits included increased job satisfaction, the ability to provide holistic care, increased knowledge, confidence and decision-making skills, alongside enhancements to their professional role to develop into well informed practitioners capable of treating the plethora of medical conditions encountered in practice. While an increased range of medication was also cited as a potential benefit of PISP, advanced paramedics adopting PISP will develop a personal formulary and only prescribe within their scope of knowledge and confidence (College of Paramedics, 2018).

The majority of SPEUCs in this study also held views which were in accord with findings from research regarding NISPs and that the introduction of PISP might also involve negotiating several challenges. These included a lack of organisational readiness, difficulties accessing patient records to support prescribing, increased responsibility, risk of litigation and professional consequences, alongside difficulties accessing supervision from the medical profession. Peer support for prescribing post-qualification has also been found to be highly important to NISPs’ confidence (Courtenay et al., 2012; Herklots et al., 2015; Latter et al., 2010).

SPEUCs also anticipated a lack of organisational support for PISP. This has also been reported by NISPs and that nurse independent prescribing is often driven by individual practitioners rather than at an organisational level (Courtenay et al., 2012; Latter et al., 2010; Stenner et al., 2012).

These findings highlight how, in the period leading up to implementation of PISP, ways of enabling access to both patient records and professional prescriber support will need to be addressed in order to support safe and confident prescribing by advanced paramedics.

This study has some important limitations. It is acknowledged that a response bias may exist in this study and the small sample size may not be fully representative of the SPEUC population. Respondents who participated may have been those who were more favourably disposed towards PISP.

## Conclusions

The use of PGDs enable SPEUCs to deliver important treatment to patients in the community and thus potentially enable them to contribute to a reduction in the number of patients requiring hospital treatment. They were also reported to provide a safe and frequently effective method of medication supply. Nonetheless, it would seem that due to the highlighted restrictions associated with a reliance on PGDs as a primary method of SPEUC medication supply, both patient care and the professional role of SPEUCs will be enhanced through the introduction of PISP. However, for PISP to be successfully introduced a number of contextual issues will need to be addressed, such as providing support and improving organisational readiness.

## Acknowledgements

The authors would like to acknowledge the hard work of and assistance from the staff in both participating ambulance Trusts and thank them for their help during the study.

## Author contributions

AMB and SML were equally involved in the design of the pilot and final questionnaire, data analysis, interpretation and in writing this paper.

## Conflict of interest

None declared.

## Data sharing

Data from this study are held securely by the University of Southampton and are not available to be accessed.

## Ethics

Ethical approval was granted from the University of Southampton Faculty of Health Sciences Ethics Committee (ERGO ID 20044). The study was also approved by the Health Research Authority (HRA) (IRAS ID 205342).

## Funding

AMB received funding from the National Institute for Health Research (NIHR) to complete a Master of Research (MRes); this study was completed as an empirical dissertation project for this MRes.

## Patient consent

Not applicable.
